# The hemogenic endothelium: a critical source for the generation of PSC-derived hematopoietic stem and progenitor cells

**DOI:** 10.1007/s00018-021-03777-y

**Published:** 2021-02-09

**Authors:** Lucas Lange, Michael Morgan, Axel Schambach

**Affiliations:** 1 Institute of Experimental Hematology, Hannover Medical School, 30625 Hannover, Germany; 2 REBIRTH, Research Center for Translational Regenerative Medicine, Hannover Medical School, 30625 Hannover, Germany; 3 Division of Hematology/Oncology, Boston Children’s Hospital, Harvard Medical School, Boston, MA 02115 USA

**Keywords:** Hematopoietic stem cells, Induced pluripotent stem cells, Hemogenic endothelium, Endothelial-to-hematopoietic transition

## Abstract

In vitro generation of hematopoietic cells and especially hematopoietic stem cells (HSCs) from human pluripotent stem cells (PSCs) are subject to intensive research in recent decades, as these cells hold great potential for regenerative medicine and autologous cell replacement therapies. Despite many attempts, in vitro, de novo generation of bona fide HSCs remains challenging, and we are still far away from their clinical use, due to insufficient functionality and quantity of the produced HSCs. The challenges of generating PSC-derived HSCs are already apparent in early stages of hemato-endothelial specification with the limitation of recapitulating complex, dynamic processes of embryonic hematopoietic ontogeny in vitro. Further, these current shortcomings imply the incompleteness of our understanding of human ontogenetic processes from embryonic mesoderm over an intermediate, specialized hemogenic endothelium (HE) to their immediate progeny, the HSCs. In this review, we examine the recent investigations of hemato-endothelial ontogeny and recently reported progress for the conversion of PSCs and other promising somatic cell types towards HSCs with the focus on the crucial and inevitable role of the HE to achieve the long-standing goal—to generate therapeutically applicable PSC-derived HSCs in vitro.

## Introduction

Definitive bona fide hematopoietic stem cells (HSCs) are defined based on specific and unique hallmarks of self-renewing cells with long-term engraftment and full multi-lineage reconstitution potential after transplantation in a conditioned recipient. Postnatally, HSCs reside in specialized bone marrow (BM) niches that preserve (1) HSC in a multipotent, self-renewing steady state or (2) facilitate differentiation into mature progeny via asymmetric cell divisions. HSCs form the apex of the hierarchical scheme of adult hematopoiesis and give rise to hematopoietic progenitor cells (HPCs), which, in contrast to HSCs, are characterized by limited self-renewal, engraftment and lineage potential. HSCs provide a constant supply of all hematopoietic cells throughout the entire lifetime of an organism. These hallmarks make HSCs an invaluable cell source and HSC transplantation has become a standard for cell replacement therapy to treat a variety of hematological diseases and malignancies [1, 2]. While murine HSC ex vivo expansion is well established, ex vivo long-term expansion of functional human HSCs is still challenging [3]. This poor ex vivo expansion leads to relatively low quantity and quality of functional human HSCs. Furthermore, immunological incompatibilities are another limiting factor for the use of HSCs for transplantation and necessitate human leukocyte antigen (HLA) matching between donors and recipients [1, 2].

Advances in the cultivation, generation and differentiation of pluripotent stem cells (PSCs) and especially the reprogramming of somatic cells into induced pluripotent stem cells (iPSCs) [4, 5] would overcome many of these limitations and represent a potential paradigm shift in regenerative medicine. Generally, iPSCs are generated by ectopic expression of the transcription factors (TFs) *OCT4*, *SOX2*, *MYC* and *KLF4* [4] in somatic and well-accessible cells. iPSCs have an indefinite proliferation potential in culture and the capacity to be differentiated into all somatic cell types. These properties offer a potential use of the iPSC technology for personalized and autologous cell-based therapies in a variety of diseases. Improved genome editing technologies further enhance the potential use of iPSC as powerful tools in basic research, disease modeling, drug screening as well as to mimic ontogenetic and pathophysiological processes in vitro [6]. However, the clinical utility of iPSC-derived cell products is heavily dependent on several factors, including the differentiation techniques, cost-effective scale-up to produce adequate numbers of therapeutic cells and, most strikingly, on the safety and functionality of the final cell product.

Despite these advances and vigorous research over the last decades, de novo generation of PSC-derived functionally transplantable HSCs in vitro remains challenging and a high priority for hematology and regenerative medicine. PSC-derived functional HSCs generated under experimental conditions had reconstitution and engraftment potential as shown by in vivo teratoma formation approaches [7, 8], providing evidence for the HSC capacity of PSCs. However, these approaches highly rely on specific, instructive niches and cell–cell interactions and are far from defined conditions. Two major approaches are predominantly used for in vitro differentiations: (1) use of defined cell-extrinsic factors for directed differentiation (e.g., defined morphogens, serum, conditioned media or co-culture systems) and/or (2) direct conversion and forward programming through TF-mediated cell fate determination. Both strategies rely on recapitulating crucial aspects of ontogenetic processes and require a detailed understanding of critical stages of early hemato-endothelial development. To more thoroughly explore these different strategies, it is important to first discuss primitive and definitive hematopoiesis.

## Embryonic hematopoiesis in mammals: drawing lessons from development

**Fig. 1:**
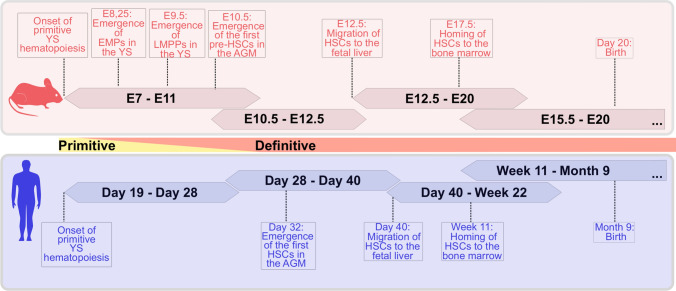
Simplified two-stage model of the spatiotemporal organization of embryonic hematopoiesis in mice and humans. Scheme of the timing and emergence of hematopoietic cells during hematopoietic ontogeny of mice (in red) and humans (in blue). Primitive hematopoiesis is the initial wave in the extraembryonic yolk sac (YS), followed by the emergence of definitive erythro-myeloid progenitors (EMPs) and lymphoid-primed progenitors (LMPPs) in the extraembryonic compartment. The first HSCs arise in the intraembryonic aorta–gonad–mesonephros region (AGM). The AGM-derived, immature, pre-HSCs migrate and colonize the fetal liver for a maturation and expansion step. After this expansion, the mature HSCs mobilize to the bone marrow, where they reside throughout the adult life after birth

The hematopoietic ontogeny is complex and encompasses temporal and spatial patterns. These spatiotemporal differences are most commonly represented as a simplified two-stage model of successive waves of primitive (first wave) and definitive (second and third waves) hematopoiesis, that differ in their hematopoietic potential (Fig. 1).

### Primitive hematopoietic wave

The primitive wave is considered as the initial program of embryonic hematopoiesis. Shortly after mesodermal formation, cells of the primitive streak form the extraembryonic yolk sack (YS) and the vascular plexus, containing the blood islands [9]. The primitive hematopoietic program is initiated between embryonic day 7 (E7) and E8.5 within the blood islands of the mouse embryo [10] and during the third week in the human ontogeny [11] (Fig. 1). This hematopoietic wave is highly restricted, with the primary function to produce primitive erythrocytes, macrophages [10] and megakaryocytes [12], independent of HSCs.

### Definitive hematopoietic waves

In the mouse embryo, between E8.25 and E10, YS hematopoiesis also gives rise to multipotent progenitors, with definitive erythrocytes, megakaryocytes and granulocyte–macrophage progenitors, and is broadly termed EMP-hematopoiesis (erythro-myeloid hematopoiesis) as the second wave of hematopoiesis [10, 13, 14]. In the later stage of the second wave of murine hematopoiesis and overlapping with the EMP-hematopoiesis, extraembryonic YS hematopoiesis also gives rise to multipotent progenitors with lymphoid (NK, B and T cell) potential [15–17]. Based on their lymphoid potential, these progenitors have been named lymphoid-primed progenitors (LMPP) (Fig. 1).

This transient second hematopoietic wave produces multipotent progenitors (EMP, LMPP) with several blood lineages and definitive erythrocytes, independent of the primitive hematopoietic wave. Therefore, this wave can be considered as the onset of definitive hematopoiesis [18, 19], although the origin of these hematopoietic cells is the YS and prior to the activity of HSCs. Lineage-tracing studies further provided evidence for the HSC-independent lymphoid progenitor potential of the YS. These progenitors demonstrated lymphoid and myeloid potential, but lacked erythro-megakaryocytic potential and were traced back to E9.5 in the extraembryonic YS. These data suggest the existence of a lympho-myeloid progenitor that precedes HSC development [20, 21]. In contrast to the murine embryo, de novo generation of YS-derived LMPPs was not observed before the onset of circulation in the extraembryonic YS during human hematopoietic ontogeny [22, 23]. This further indicates significant evolutionary differences between human and mouse embryonic hematopoiesis.

The defining quality of the third wave of hematopoiesis is the generation of bona fide HSCs with the capacity to engraft adult recipients.

### Embryonic origin of HSCs

Transplantation experiments of cells acquired from different developmental stages of murine hematopoietic cells showed that the first occurrence of definitive HSC with the capacity to engraft adult recipients arise between E10.5 and E11.5 [24] and in the human embryo at day 32 of gestation [25] (Fig. 1), independently of the YS hematopoiesis [26]. At this time point, a splanchnopleural mesoderm-derived, intraembryonic, definitive hematopoietic site was identified as the aorta–gonad–mesonephros (AGM) [27, 28] region, particularly the dorsal aorta (DA) [29–31], which is probably the best-studied site for de novo HSCs generation. Although the DA is an origin for HSC emergence, the numbers of HSCs in the AGM region are low [32–34], and HSCs are only present transiently at this site. Therefore, the AGM is not considered to be a major site for HSC expansion. Shortly after HSC emergence, AGM-derived HSC migrate and colonize different fetal hematopoietic sites, where they mature and expand. In mouse, cells with multi-lineage repopulation activity were first detected in the fetal liver (FL) at E12, concomitant with a dramatic expansion and formation of an FL HSC pool [35], until mobilization of HSCs out of the FL towards other hematopoietic tissues like thymus and finally, the bone marrow [36]. Although several different sites with hematopoietic activity have been described during mammalian ontogeny, the primary origin(s) of hematopoietic cells and, in particular, the embryonic ancestor of HSCs remain controversial and a current area of extensive research.

## The hemogenic endothelium: an endothelial link to hematopoietic development and the womb of definitive HSCs

More than 100 years ago, Sabin observed aggregates of hematopoietic cells budding from a layer of endothelial cells in chick embryos [37]. This observation and the concomitant temporal and spatial emergence of endothelial and hematopoietic cells during vertebrate ontogeny led to the hypothesis of a close developmental correlation between endothelial and hematopoietic cells. This hypothesis was further supported and validated in later experiments. Ex vivo culture of murine KDR^+^ (kinase insert domain-containing receptor; vascular endothelial growth factor receptor 2) endothelial cells gave rise to multi-lineage hematopoietic cells with reconstitution potential after intrahepatic injection into conditioned newborn recipient mice [38]. Lineage-tracing studies tracked the fate of CD144^+^ endothelial cells that gave rise to multi-lineage hematopoietic cells in vivo [39]. Time-lapse confocal imaging of murine E10.5 DA validated the endothelial origin and showed a dynamic emergence of hematopoietic cells, directly sprouting from ventral aortic endothelial cells [40]. These elegant experiments demonstrated that hematopoietic cells, including HSCs, arise through an intermediate endothelial state known as hemogenic endothelium (HE).

### HE surface markers: murine and human

**Fig. 2:**
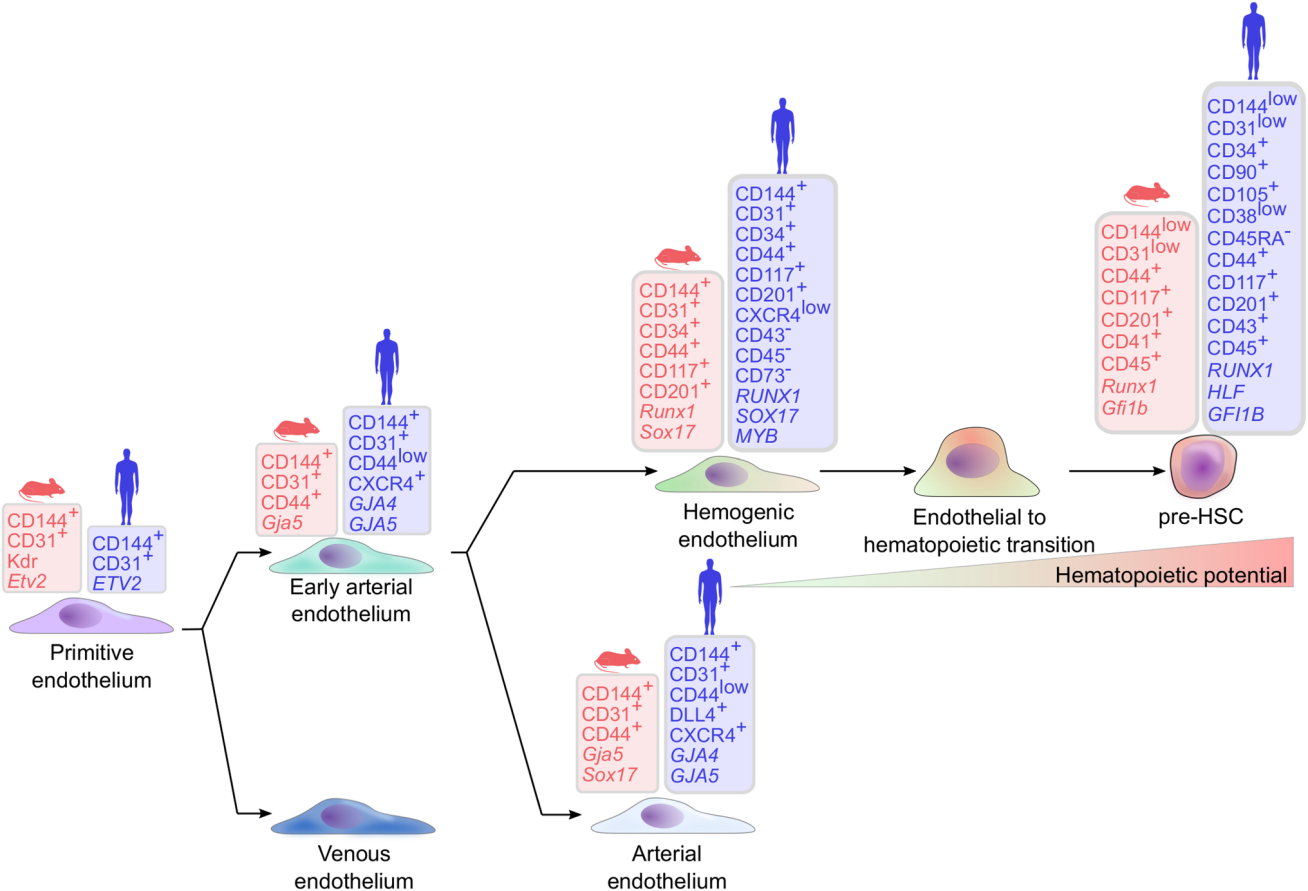
Simplified model of HSC emergence through different intermediate endothelial stages in mice and humans. Two major fate decisions precede HSC emergence in a dynamic process. Primitive endothelial cells first acquire an early arterial fate, followed by hemogenic endothelial specification, segregated from mature arterial endothelium. The early arterial endothelium-derived hemogenic endothelium (HE) gives rise to pre-HSCs through a gradual endothelial to hematopoietic transition. All intermediate developmental stages can be segregated based on their functionality and different gene expression and surface marker profiles. The phenotype of the different developmental stages is based on a combination of PSC differentiation, in vivo lineage tracing and single cell transcriptome fate mapping. Figure includes data from references [31, 41–49, 64, 71, 73, 78, 112]

By definition, HE is a transient, specialized endothelium with the capacity to generate hematopoietic cells through a gradual process of endothelial-to-hematopoietic transition (EHT) [41]. So far, no unique surface marker has been described to identify HE. Murine endothelium with hemogenic potential are generally identified retrospectively by the potential to give rise to hematopoietic cells and are often characterized by co-expressed surface markers CD144, CD31, KDR, CD117, CD34, and the lack of hematopoietic-associated markers such as CD41, CD45 and Ter-119 [42, 43] (Fig. 2). A similar immunophenotype was also found on human PSC-derived HE with co-expression patterns of surface markers CD144, CD31, KDR, CD117 and CD34 and lack of CD43 [44–46]. In combination with these markers, the lack of CD73 expression was identified to demarcate endothelium with hemogenic potential from non-hemogenic endothelium. During the transition from endothelial cells towards a hematopoietic cell type, endothelial cells gradually lose endothelial characteristics, and concomitantly acquire a hematopoietic phenotype and morphology [41, 47]. In humans, the early emerged hematopoietic committed cells can be identified based on the surface markers CD43, CD34, CD144, CD117, CD90, CD45, CD105, low CD38, and the lack of CD45RA (Fig. 2) [31, 46, 48, 49].

Although the concept of the HE as a precursor of hematopoietic cells is best studied in the AGM region, several other endothelial sites with hemogenic potential have been described in the YS, placenta, major arteries (umbilical and vitelline arteries) and head [30, 50–57]. It was shown that lymphoid cells and, more strikingly, HSCs are predominantly derived from arterial-type hemogenic endothelium [58–61]. However, are all of these hemogenic cells identical, or are there functional, transcriptional, and/or developmental heterogeneities among HE cells?

### Signal transduction and gene expression patterns in HE to HSC transition

Notch signaling has a central role during HSC emergence, endothelial development, and arterial identity of the endothelium [62–67]. Notch knockout studies in zebrafish demonstrated that primitive hematopoiesis is independent of Notch signaling. Definitive hematopoiesis and HSC emergence necessitated Notch signaling and were linked to *runx1* expression as a direct downstream target [68]. Similarly, HE demonstrated pre-existing arterial endothelial characteristics, suggesting an arterial endothelium as a direct precursor [69]. The hematopoietic commitment of the arterialized endothelium was initiated by *runx1* expression and, as a consequence, resulted in downregulation of *runx1*-regulated arterial genes like *sox17* or the Notch ligand *dll4* in zebrafish [69]. Interestingly, the dosage of Notch signaling and the balance between Notch-Dll4 and Notch-Jag1 signaling was described to be crucial for either arterial endothelial or hemogenic cell fate in mice [70]. High Notch-signaling activity through Dll4 favors arterial endothelial specification, whereas low Notch signaling through Jag1 activates hematopoietic genes and commitment [70]. In the same study, Jag1-induced microRNA expression was described, which might posttranscriptionally regulate the endothelial-associated gene expression [70].

Single-cell RNA sequencing of different developmental stages of the human AGM validated the arterial origin and mapped the developmental fate of HSCs through an arterial endothelium, and an intermediate arterialized HE [71]. This approach identified an *ETV2*-expressing endothelial precursor, which independently gave rise to both arterial and venous endothelium with distinct hemogenic potential. The human AGM region HE exhibited typical expression of genes, associated with arterial-type endothelium (e.g., *GJA5*, *GJA4*, *HEY2*, *CXCR4*, *DLL4*, *MECOM*, and *HES4*), including crucial genes of the NOTCH-signaling pathway and was almost entirely absent of venous characteristics. Along with arterial HE differentiation, expression levels of *EMCN*, *RUNX1T1* and *PROCR* were increased, which decreased upon hematopoietic commitment, concomitant with upregulation of *PTPRC*, *ANGPT1* and *SPINK2* in emerging HSCs [71]. Interestingly, the same study identified the surface marker CD44, a marker previously described to be expressed in the inner layer of endothelial cells in the DA [72], to be almost explicitly expressed on arterial endothelial cells with hemogenic potential, but seldom on venous HE [71]. Thus, CD44 might be a suitable marker to characterize the developmental stages and identify arterialized HE and HSC emergence for in vitro differentiation. In line with this approach, single cell transcriptome analyses used to map the fate of endothelial cells towards hematopoietic cells in mid-gestational mice AGM regions between E9.5 and E11 similarly demonstrated an early arterial endothelial precursor of HE [73]. Computational prediction of their single-cell RNA-sequencing data revealed two bifurcations and fate decisions of endothelial cells during HE specification, which were distinguishable by their gene expression (Fig. 2). The first fate decision occurred in primitive endothelial cells between a venous endothelial phenotype and a primitive arterial-type endothelium. Later, the early arterial endothelium acquired either a mature arterial phenotype (late arterial endothelial cells) or became HE with the capacity to generate committed HSC precursors (pre-HSC) [73] (Fig. 2). Pre-HSCs can be subdivided into pro-HSCs, type I pre-HSCs and type II pre-HSCs based upon their maturation stage and engraftment capability [61, 74–76]. Surprisingly, the same study described a bi-potent rare, putative committed pre-HSC capable of endothelial and hematopoietic specification [73, 77]. This finding underlines the dynamic progress of this transition and raises the question at which time point the final commitment of pre-HSCs occurs. Similar to the transient upregulation of *PROCR* (also known as EPCR or CD201) expression upon hemogenic fate specification in the human AGM [71], CD201 marked murine HE populations and pre-HSCs [73, 78] and could be a putative marker for in vitro-derived HSC-primed, arterialized HE. Interestingly, EPCR was further found to mark engraftment- and reconstitution-competent HSCs derived from human cord blood CD34^+^ cells that were expanded with UM171 [79].

### Emergence of HSCs from the HE

**Fig. 3:**
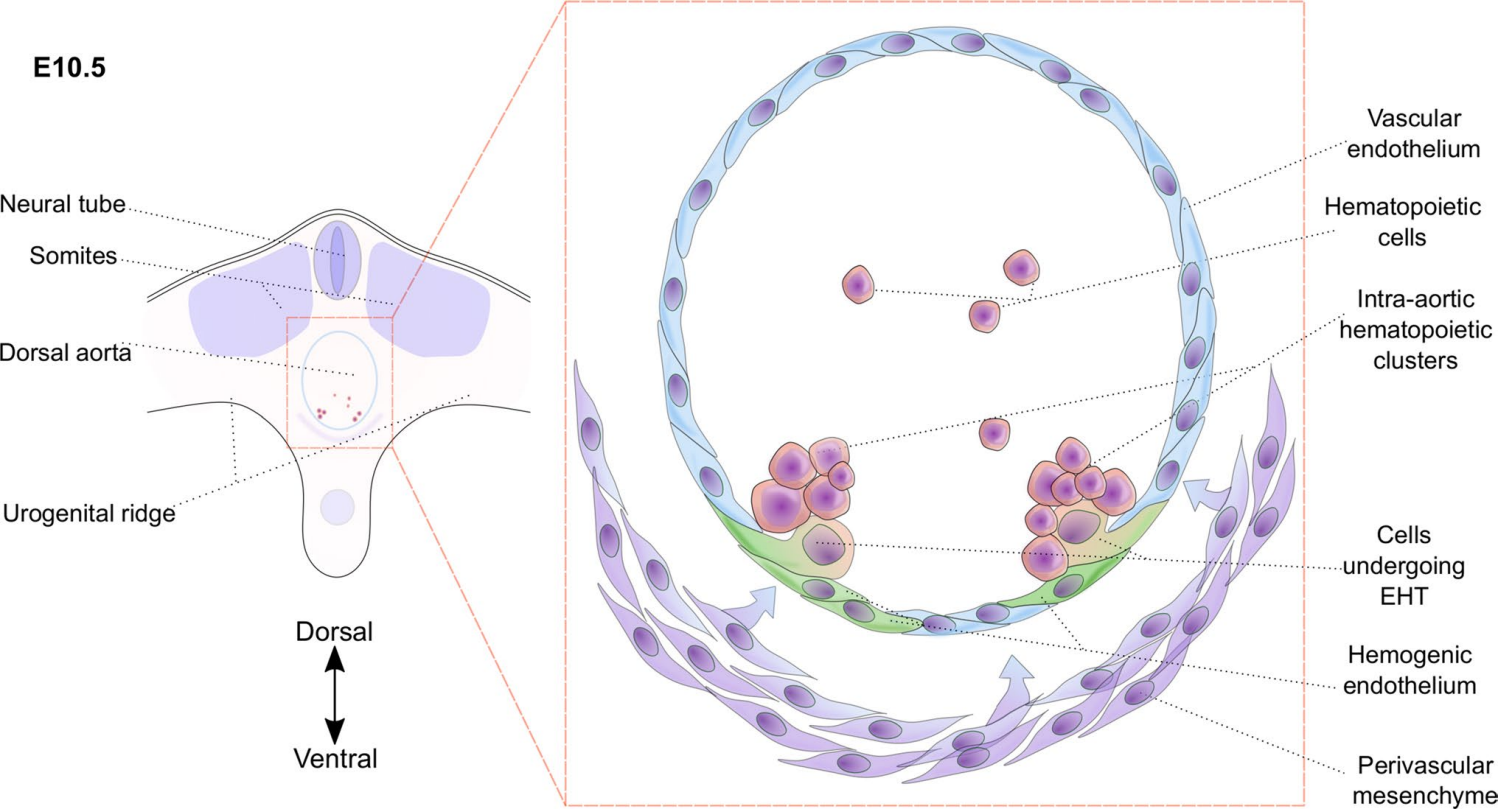
Hemogenic fate specification and HSC emergence from hemogenic endothelium (HE). **a** Schematic cross-section through the murine E10.5 aorta–gonad–mesonephros (AGM) region. The dorsal aorta (DA) is boxed (red dashed box) and magnified to visualize the cell types involved in AGM hematopoiesis. Hematopoietic cells, including pre-HSCs, arise through an intermediate hemogenic endothelium (green), organized in intra-aortic hematopoietic clusters (IAHCs). This endothelial-to-hematopoietic transition (EHT) and HSC emergence is regulated and directly or indirectly influenced by signaling and cell-extrinsic factors from the microenvironment (e.g., vascular endothelial cells (light blue) and perivascular mesenchyme (purple))

In vivo, the vast majority of the endothelial cells within the hematopoietic sites during ontogeny are vascular endothelial cells without hemogenic potential. Only small subsets of endothelial cells demonstrate the capacity for de novo hematopoietic cell generation. During murine embryogenesis, hematopoietic cells arise through an intermediate HE between E7.25 [55] to shortly after birth [80]. During the EHT process in the DA of the E10.5 AGM, endothelial cells and the derived hematopoietic cells are organized in clusters, attached to an endothelial layer and bud into the lumen of the vessel (Fig. 3) [81–83]. For the DA, these clusters are later referred to as intra-aortic hematopoietic clusters (IAHCs) and the formation is highly conserved and described for several vertebrate species [30, 84, 85], including humans [49]. Not all hematopoietic cells that arise in the AGM region through IAHCs are bona fide mature HSCs. In mouse, IAHCs consist of an HSC precursor (type II pre-HSC) and already committed hematopoietic progenitors. However, in mice, the majority of the IAHC probably comprise very immature pre-HSC (type I or pro-HSC), yet incapable of long-term engraftment or multi-lineage reconstitution of neonate recipients [74–76], but able to progressively mature towards bona fide HSCs within different hematopoietic sites or neonatal environments [34, 58, 86]. While IAHCs are also formed within lateral, dorsal and ventral endothelial layers, preferentially the ventral section had the autonomous capacity to generate HSCs with reconstitution potential [29]. This implies a dorso-ventral polarity of HSC generation and indicates a putative functional heterogeneity among IAHCs and, consequently, HE.

Astonishingly, the presence and functional activity of pre-HSCs have also been shown in the dorsal domain of the DA [87]. In accord with these findings, RNA-sequencing comparison of murine dorsal IAHCs and ventral IAHCs between E10 and E11 revealed only minor differences in the transcriptome [75]. However, the most dramatic transcriptional changes were observed mainly in the ventral IAHCs during the EHT process, as well as during formation and maturation of pre-HSCs [75]. This indicates an instructive role of anatomically distinct environments and, as a consequence, differential influences of signaling and cell-extrinsic factors on HE and IAHCs. Interestingly, the expression of hematopoietic-associated genes such as *Runx1* and *Gata3* was described in murine CD45^−^ mesenchymal cells, which are located ventral to the DA [88–91]. This led to the hypothesis of a putative different and direct origin of HSCs and it has been speculated whether the sub-aortic mesenchymal floor of the AGM has an instructive role or directly gives rise to pre-HSCs. In vivo*,* lineage-tracing studies in the murine AGM indicated that this sub-aortic mesenchyme was not a direct progenitor of HSCs [39]. Some evidence suggests that the early, transient, lateral plate mesodermal-derived, mesenchymal population may contribute to the aortic floor endothelium [92], which, in turn, has the capacity for hematopoietic cell generation through an intermediate endothelium [39]. However, the role of the sub-aortic mesenchyme is still a matter of controversy in the field, which remains to be resolved [93].

The sub-aortic mesenchyme potentially provides an instructive microenvironment and signaling that supports EHT and, subsequently, HSC emergence. Elegant ex vivo studies of mouse E10.5 AGM region identified that interactions among three main signaling pathways favor HSC emergence in the ventral domain of the DA [87]. Overlapping gradients and asymmetric patterns of (1) urogenital ridge and ventral DA domain-derived stem cell factor (Scf), (2) sonic hedgehog (Shh) produced in the dorsal domain of the DA and (3) ventral Bmp4 inhibition through Noggin expression were found to be essential for the generation of HSCs in the ventral DA domain [87]. Similarly, different key signaling pathways were described to be indispensable for early mesodermal patterning and hemato-endothelial ontogenesis in animal models and that precise and spatiotemporal regulation of these pathways is critical. Early during zebrafish embryonic hematopoiesis, Bmp4 signaling was described to promote hematopoietic specification from mesoderm, mainly through induction of Wnt and, as a consequence, upregulation of caudal-related homeodomain (Cdx) TFs [94]. In mice, Cdx1 and Cdx4 are directly regulated by Wnt signaling [95, 96] and Cdx genes have been further described to control cell fate determination through *Hox* gene regulation [97]. These key signaling pathways are mostly conserved among vertebrates and have been exploited to direct in vitro hematopoietic differentiation. All of these pathways act as a dynamic, complex network of interacting signaling cascades to precisely mediate control of developmental stages and cell fate decisions.

Although there is remarkable evolutionary conservation among vertebrate genomes, considerable genetic differences between human and other vertebrate species have been observed, which contribute to crucial dissimilarities during embryonic hemato-endothelial development. These differences can be significant and might preclude the transfer of developmental concepts and regulation from model organisms to human developmental processes. Therefore, the use of human cells and especially differentiation of human PSCs emerged as a powerful tool to mimic and investigate human developmental processes and their regulation in vitro. However, many of the developmental concepts observed in model organisms, the cell-extrinsic and -intrinsic regulation of hemato-endothelial development, have been used to design successful hematopoietic differentiation protocols of human PSCs in vitro. The insights of these PSC-based hematopoietic differentiations can be used to validate the in vivo findings and complement and shape our knowledge about human hematopoietic ontogeny.

## Directed differentiation of PSCs towards hematopoietic cell types

Directed differentiation is based on recapitulating and mimicking key aspects of embryonic hematopoiesis and the regulation of ontogenetic processes in vitro by instructive cell-extrinsic factors [45, 98–105]. Directed hemato-endothelial differentiation of PSCs has been explored for decades [100, 106] and paved the way for the upcoming differentiation protocols. While many current directed differentiation protocols rely upon well-characterized serum-free medium components, there are still some less well-defined culture components such as factors derived from co-cultivation systems. In addition, it is difficult to quantify effects of cell-extrinsic factors like cell–cell interactions on in vitro differentiation. This limits accurate control and reproducibility of the differentiation process and has thus far only produced a limited range of mature hematopoietic lineages and HPCs without long-term reconstitution potential. Thus, it is likely that directed hematopoietic differentiation rather resembles the first, transient, HSC-independent waves of embryonic hematopoiesis. Usage of more defined, serum-free media, defined morphogens, small molecules and culture conditions enables the production of hematopoietic cells and HE in a more defined and reproducible manner. However, the generation of PSC-derived, bona fide HSCs under in vitro conditions remains a significant challenge and is still a high priority in the fields of hematology and regenerative medicine. These restrictions are probably due to the lack of detailed understanding of human hemato-endothelial ontogeny and the limitations of recapitulating complex, dynamic, multifactorial developmental processes in vitro.

Although the clinical use of PSC-derived HSCs remains to be achieved, in vitro hematopoietic differentiations undoubtedly contributed to our current understanding of early human hematopoietic development. More importantly, early experiments provided compelling evidence that crucial stages of human ontogeny can be modeled in vitro. Several studies have convincingly demonstrated that primitive and definitive hematopoietic cells arise through specialized endothelial cells with hemogenic capacity [45, 55, 107–111]. Mostly, directed differentiations generate different subtypes of mesodermal progenitors and cells with different hemogenic or vascular endothelial potential. Choi et al*.* identified hemogenic endothelial cells based on the immunophenotype CD144^+^/CD73^−^/CD235a^−^/CD43^−^. This HE gave rise to HPCs with an enhanced myeloid and erythroid lineage potential [45], but, more importantly, they neatly dissected hemato-endothelial specification from human PSCs and identified populations of cells with distinct endothelial and hematopoietic potential [45]. This indicates the simultaneous emergence of transient primitive and definitive hematopoietic programs in vitro and potential functional heterogeneity of the hemogenic capacity of the endothelial cells. Single cell transcriptional analysis of human iPSC-derived CD34^+^ cells confirmed the functional heterogeneity. Transcriptional stages of HE cells (CD34^+^/CD43^−^/CD90^+^/CD73^−^/CXCR4^−^) during the narrow window of the EHT process were dissected and were used to identify sub-populations with distinct hematopoietic lineage potential [112]. Based on these findings, it was hypothesized that the distinct hematopoietic lineage capacities are defined within the cell populations at the EHT stage, and therefore, before the complete loss of endothelial characteristics [112].

A different study proposed that distinct hematopoietic potential is already determined during mesodermal patterning. The erythroid surface marker CD235a was surprisingly found to be expressed on mesodermal, KDR^+^ precursor cells, fated to the primitive hematopoietic lineages [105]. In contrast, the KDR^+^/CD235a^−^ mesodermal population could generate a broader spectrum of mature hematopoietic lineages, including T-lymphoid cells [105]. This fate determination was attributed to a dynamic interplay between the WNT signaling pathway and Activin-Nodal signaling [105] and has also been linked to the CDX-HOX pathway [113]. Similarly, modulation of the WNT and Activin-Nodal signaling pathways in mesodermal cells resulted in the upregulation of *CDX4* and, as a putative consequence, upregulation of *HOXA3*, *HOXA5*, *HOXA7*, *HOXA9* and *HOXA10* expression. Moreover, this modulation directed endothelial cells towards a SOX17^+^ aorta-like endothelial cell phenotype with hemogenic potential [114]. Although these studies demonstrated an enhanced hematopoietic potential, the definitive hematopoietic potential was measured based on the emergence of T-lymphoid cells. Generation of HSC-like cells with repopulating potential was not observed [105, 113, 114]. This suggests that the cells were either a progenitor of the transient EMP/LMPP hematopoiesis or indicated the requirement for additional, complementary regulatory factors or signaling to facilitate HSC function.

Consistent with the pivotal role of Notch signaling during endothelial development formation of the dorsal aorta and, as a consequence, HSC emergence [65–70], NOTCH-DLL1 signaling facilitates arterialization of human PSC-derived HE in vitro [64]. Interestingly, and in contrast to murine in vivo data [70], immobilized JAG1-Fc had only minor effects on hematopoiesis. Activation of NOTCH signaling through immobilized NOTCH-ligand DLL1-Fc in CD31^+^ (PECAM1, an endothelial-specific marker) cells led to the upregulation of typical NOTCH-downstream genes (*HES1*) and expression of typical arterial-associated genes (e.g., *DLL4*, *EFNB2*, *HEY2*, *SOX17*, and *CXCR4*) in a transient, CD144^+^/CD73^−^/CD43^−^/DLL4^+^ HE population. This HE had the capacity to undergo EHT and produce lymphoid, myeloid and erythroid cells in a NOTCH-dependent manner. In contrast, the non-arterialized HE population (CD144^+^/CD73^−^/CD43^−^/DLL4^−^), showed mostly primitive hematopoietic potential. Although the arterialized HE was able to give rise to definitive lympho-myeloid hematopoietic cells, these cells were not engraftment-competent HSCs. Most strikingly, the arterialized HE had only the capacity to generate hematopoietic cells in co-culture with OP9-DLL4 cells, but not under defined, serum-free conditions. Thus, additional, unknown, stroma-cell derived factors were crucial for the EHT process, which activates or inhibits different signaling pathways.

The overall mode of action of signaling pathways is mostly similar. A cell-extrinsic signal is converted into a cellular response through intracellular signaling cascades and usually results in gene expression changes. TFs are often direct targets of signaling cascades, which directly alter the transcriptional response and, subsequently, downstream regulation of associated genes and transcriptional networks. Overexpression of these downstream TFs might bypass or provide shortcuts to complex cellular processes, cell–cell interactions and signaling cascades and might help to simplify demanding differentiation protocols and improve hemato-endothelial differentiation processes.

## Transcription factor-mediated enforced hematopoietic specification

**Fig. 4:**
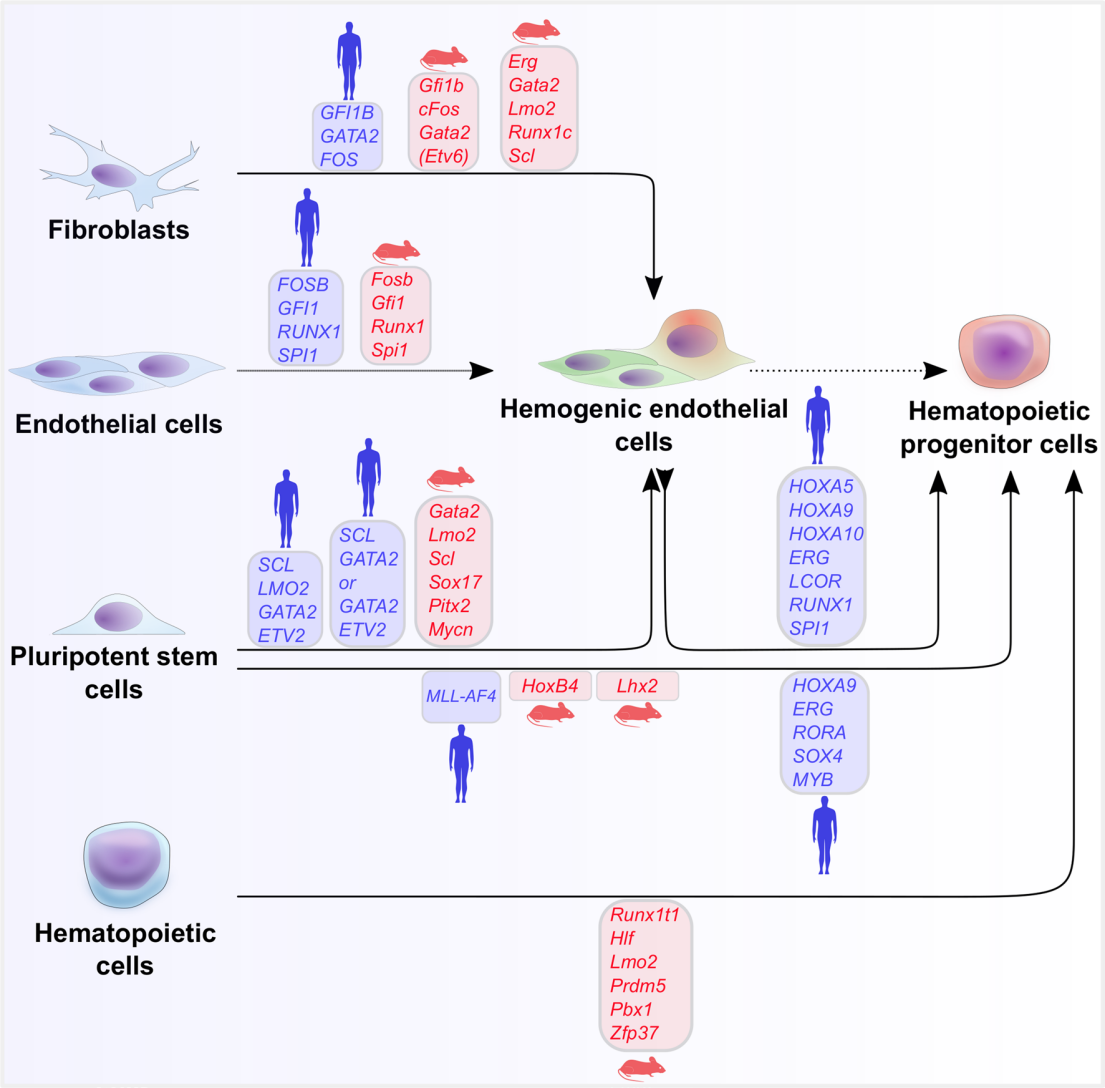
Transcription factor-mediated hematopoietic differentiation strategies. Hematopoietic in vitro differentiation approaches based on the ectopic overexpression of transcription factors (TFs) in somatic cell sources, such as fibroblasts, mature non-hemogenic endothelial cells, lineage-committed hematopoietic cells or pluripotent stem cells, such as embryonic stem cells (ESCs) or induced pluripotent stem cells (iPSCs). The used TFs/TF combinations are shown in boxes and color-coded in red for murine cell origin and blue for human cell origin

Alternative approaches have emerged to overcome the limitations of directed hematopoietic differentiation strategies by the generation of HE, hematopoietic cells, or even HSC-like cells through ectopic expression of cell fate-determining TFs [115, 116]. These TFs can either be overexpressed in (1) mature cell types for direct conversion into less committed intermediate precursors, or (2) PSCs for forward programming into specific lineages (Fig. 4 and Table 1). The identification of master regulators and, more importantly, interacting TF combinations and transcriptional networks is crucial for both strategies. Many TFs have been used in in vitro differentiation approaches based on their described key roles during vertebrate ontogeny in vivo.

**Table 1:**
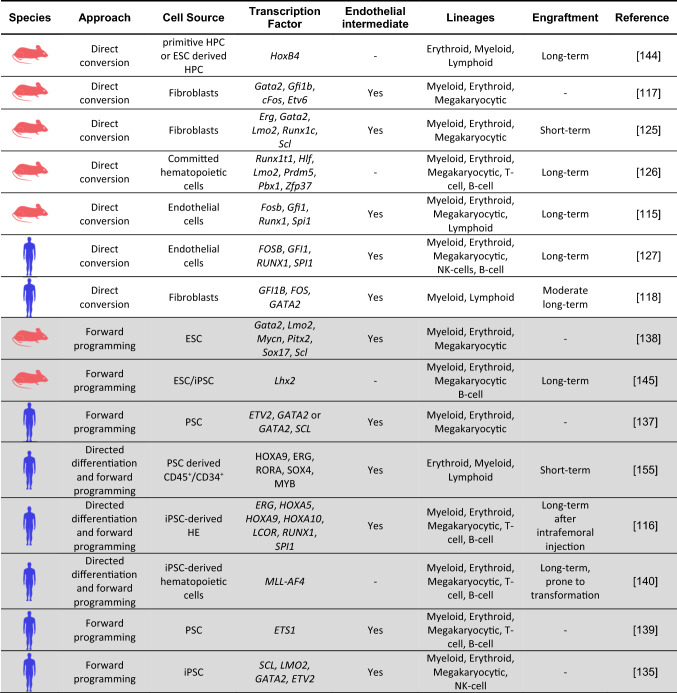
TF-mediated hematopoietic differentiation approaches of murine and human cell origin. PSC-based approaches marked in light grey. Information modified and extended from Wahlster and Daley [157]

Expression of the TF combination *Gata2*, *Gfi1b*, and *cFos* (enhanced with *Etv6*) induced hematopoietic potential in murine fibroblasts. The transduced fibroblasts formed endothelial-like structures and produced hematopoietic cells in a dynamic process through a Tie2^+^ CD144^+^ CD31^+^ endothelial intermediate [117]. Similarly, ectopic expression of these TFs (*GATA2*, *GFI1B* and *FOS*) was later used for initial induction of endothelial signature, followed by hematopoietic gene expression in human fibroblasts [118]. The hematopoietic cells arise through an endothelial intermediate and demonstrated an HSC-like immunophenotype of CD34^+^/CD49f^+^/CD90^+^/CD38^−^/CD45RA^−^ [118], similar to the phenotypic definition of human cord blood HSCs [119, 120]. Most strikingly, these cells demonstrated moderate multi-lineage reconstitution potential in NSG mice up to 12 weeks post-transplantation [118]. While GATA2, GFI1B and FOS form a transcriptional complex that initiates expression of endothelial and hematopoietic genes, GATA2 was described to be the dominant transcription factor in this complex [114].

The conserved function of Gata2/GATA2 in mice and humans indicates the cooperative, dominant and instructive role of Gata2/GATA2 for induction of hemato-endothelial programs. In vivo, conditional knockout of *Gata2* cis-regulatory elements in the murine AGM region resulted in diminished *Scl* and *Runx1* expression and abolished HSC generation from HE [121]. *Gata2* knockouts in CD144^+^ endothelial cells resulted in similar effects, along with lack of IAHC formation in the murine DA and HSC generation [122]. In vitro differentiation of human embryonic stem cells (ESCs) suggested that GATA2 is crucial for the EHT process [123], likely due to transcriptional regulation of downstream targets. In mice, the *Runx1 cis*-regulatory element (+ 23 Runx1 enhancer) contains Gata and Ets motifs that regulate transcription [124]. Further, a transcriptional complex of Gata2, Fli1 and Scl was found to be recruited to the *Runx1 cis*-regulatory element, which placed the key hematopoietic TF Runx1, directly downstream of these TFs [124]. Overexpression of some of these TFs (Erg, Gata2, Lmo2, Runx1c, Scl) in murine fibroblasts induced hematopoietic specification and generation of multipotent HPCs through an intermediate endothelial stage, with expression of typical endothelial markers (e.g., *Cdh5*, *Tie2*, *Pecam1*, and *Vwf*) [125]. These multipotent progenitors demonstrated an HSC-like immunophenotype, with robust erythroid, megakaryocytic and myeloid potential as well as lymphoid potential after loss of p53 function, but only short-term reconstitution ability of predominantly erythroid cells [125]. These approaches indicate that various transcriptional regulators, or even one specific TF, might be sufficient to activate and regulate similar gene regulatory networks to induce hemato-endothelial specification. However, the generation of bona fide HSCs was not achieved with these factors. This shortcoming might be attributed to an incomplete understanding of transcriptional regulation, limitations of the in vitro culture or the requirement for an instructive and supportive niche.

A screening approach identified 6 out of 36 HSC-associated transcriptional regulators to induce re-specification of committed, murine lymphoid and myeloid progenitor cells into HSCs without an endothelial intermediate [126]. Transient overexpression of these six transcriptional regulators *Run1t1*, *Hlf*, *Lmo2*, *Prdm5*, *Pbx1* and *Zfp37* was sufficient to confer HSC functionality and, most strikingly, long-term, multi-lineage reconstitution potential in primary and secondary recipients [126]. Interestingly, transient ectopic expression of these factors was sufficient to sustain the HSC functionality in vivo and stably activate gene regulatory networks that govern HSC function and identity [126].

Taking advantage of the close ontological relation between endothelial cells and hematopoietic cells, enforced expression of the four TFs *FOSB*, *GFI1*, *RUNX1* and *SPI1* reprogrammed human, non-hemogenic mature and fetal endothelial cells into self-renewing, engraftment-competent multipotent progenitors, although, with insufficient T cell potential [127]. An immortalized endothelial cell line that was previously described to support HSC expansion, likely through AKT-regulated factors, was shown to contribute to an instructive niche, which is crucial for the formation of the HE, the EHT process and the generation of multipotent progenitors [128]. More recently, overexpression of the same TFs (*Fosb*, *Gfi1*, *Runx1* and *Spi1*) and these vascular-niche-derived factors were sufficient to fully reprogram adult murine endothelial cells into HSCs with proper functionality [115]. While these approaches were mostly initiated from mature somatic cells, TF-mediated differentiation was also used to direct hemato-endothelial specification from PSCs.

In vertebrates, the ETS-family (E26 transformation specific) TFs contain approximately 30 members (e.g., FLI1, ERG, ETV2, ETV6, SPI1, and ETS1) and have been described as key TFs that regulate early vasculogenesis and hematopoietic development [129]. The ETS-family TF ETV2 (ETS variant 2) is expressed early during mesodermal formation in cells with endothelial and hematopoietic potential [130, 131] and was shown to induce expression of several endothelial- and hematopoietic-associated downstream targets [132], indicating that ETV2 governs activation of hemato-endothelial transcriptional networks. Knockout studies in mice further supported this crucial role of Etv2 during endothelial development. Etv2 ablation resulted in significantly diminished Kdr expression and early embryonic lethality due to a complete lack of endothelial and hematopoietic specification [132, 133]. In vitro differentiation experiments validated the crucial and instructive role of ETV2. Ectopic expression of ETV2 induced expression of endothelial-associated genes (e.g., *FLI1*, *ERG*, *CDH5*, *KDR*, and *PECAM1*) and, more importantly, was sufficient to directly convert human fibroblasts into functional endothelial cells [134]. Inducible overexpression of *ETV2* in human iPSC-derived, mesodermal-primed cells resulted in an almost pure population of cells with a vascular endothelial immunophenotype (CD144^+^/CD73^+^) [135]. Similarly, transient expression of exogenous *ETV2* by modified and stabilized mRNA efficiently generated functional endothelial cells with the ability to form perfused vascular networks in vivo [136]. However, overexpression of *ETV2* alone was not described to robustly induce hemogenic potential. A gain-of-function screen of hemato-endothelial-associated TFs in human PSCs revealed synergistic effects of ETV2/GATA2 or SCL/GATA2 on hematopoietic specification [137]. Both TF combinations induced a hemato-endothelial program and generated hematopoietic cells through an intermediate endothelial state with distinct hematopoietic lineage potential [137]. Using a forward programming approach of human iPSCs, controlled overexpression of the TF combination *SCL*/*LMO2*/*GATA2*/*ETV2* robustly induced hemato-endothelial specification with an almost pure population of cells with an HE-like phenotype and, subsequently, multi-lineage HPCs [135]. However, both attempts [135, 137] demonstrated restricted lineage potential (erythroid, myeloid, megakaryocytic) with lymphoid limitations and, most strikingly, without significant engraftment and reconstitution potential. Furthermore, collective overexpression of the six TFs *Gata2*, *Lmo2*, *Scl*, *Sox17*, *Pitx2* and *Mycn* directly converted murine PSCs to hemato-endothelial cells, smooth muscle cells and hematopoietic cells [138]. Downregulation of these TFs resulted in the generation of multi-lineage hematopoietic cells through an endothelial intermediate, which were, however, restricted to erythroid, myeloid and megakaryocytic lineages [138].

In concordance with the crucial role of the arterial identity of definitive HE in vivo [59–62, 65, 67, 69, 71, 73] and the impact of arterialized HE through Notch signaling in vitro [64], overexpression of *ETS1* or modulation of the MAPK/ERK signaling pathway induced HE with arterial characteristics and enhanced lineage potential [139]. Upon ectopic *ETS1* expression at the mesodermal stage, the formation of KDR^+^/CD144^+^ endothelial cells was increased. In this endothelial population, arterial-associated genes were upregulated, including *CXCR4*, *EFNB2*, *SOX7*, *SOX17*, *SOX18* and genes of the NOTCH signaling pathway such as *DLL4*, *NOTCH1*, *NOTCH4*, and *HEY1*. The venous-specific gene *NR2F2* was not upregulated upon enforced ETS1 expression, implying an arterial-specific effect of ETS1, and suggested that HE, similar to the vascular endothelium, can acquire an arterial identity. The resulting, arterialized CD144^+^/CD43^−^/CD73^−^/DLL4^+^ HE generated an increased number of CD45^+^/CD235^−^/CD41a^−^ HPCs with erythro-myeloid and lymphoid potential. In line with a similar approach [64], the effect of the ETS1-mediated arterialization and enhanced hematopoietic potential was primarily mediated through upregulation of the NOTCH-ligand DLL4 and activation of NOTCH-mediated signaling [139]. However, both studies [64, 139] failed to achieve short- or long-term engraftment and led to speculation about the necessity for additional arterialization and NOTCH-independent mechanisms that regulate HSC specification, such as the *HOXA* gene cluster.

A variety of TFs act as master regulators and govern endothelial as well as hematopoietic ontogenesis. Transient overexpression of the MLL (mixed lineage, myeloid lymphoid leukemia)-fusion protein MLL-AF4 reprogrammed human iPSC-derived hematopoietic cells into highly engraftment-competent HSCs [140]. Although these HSCs demonstrated high levels of engraftment and reconstituted both lymphoid and myeloid lineages, the *MLL-AF4*-induced iPSC-derived HSCs were prone to leukemic transformation after transplantation [140]. In vivo, MLL is a positive regulator of *Hox* genes through direct binding to promoter sequences [141, 142] (as discussed below). Especially HOXB4 was shown to enhance self-renewal, hematopoietic capacity and, most strikingly, engraftment and repopulating potential in mice [143]. Overexpression of *HoxB4* in murine yolk sac hematopoietic progenitors or murine ESCs enabled the generation of HSC-like cells, which were able to engraft and reconstitute lympho-myeloid hematopoiesis in irradiated murine recipients [144]. Similarly, enforced expression of the LIM-homeobox TF *Lhx2* conferred long-term reconstitution potential to murine ESCs and iPSCs in primary and secondary recipient mice, however, without T-lymphoid contribution [145]. In contrast to murine ESCs/iPSCs, the repopulating capacity of human ESC/iPSC-derived hematopoietic cells was not positively affected by overexpression of *HOXB4* [146], indicating considerable differences between transcriptional regulation of human and murine hematopoietic development. Nevertheless, these studies convincingly demonstrated that overexpression of a single TF could significantly influence PSC differentiation. However, these studies mainly focused on ESC/iPSC-derived HSCs. The direct, intermediate precursor, the HE, was neglected. Later, ectopic *HOXB4* expression during the KDR^+^-stage of differentiated ESCs was associated with the promotion of HE formation [147]. The acquisition of the HE cell fate was linked to a shift of the transcriptional signature and upregulation of crucial genes and TFs for endothelial specification and hematopoiesis, such as *Cdh5*, *Cd34*, *Scl*, *Gata2*, *Erg*, *Fli1*, *Lyl1* and *Lmo2* [147]. Combinatorial expression of some of these TFs has been used to direct hemato-endothelial differentiation in several approaches.

The *HOX* genes are located in different clusters, *HOXA-HOXD*, characterized by the common homeobox DNA-binding domain [148]. The specificity and selectivity of HOX TFs are relatively low and mostly mediated and increased through co-factor binding [149]. Concomitant with the low specificity of HOX TFs, the functionality of HOX TFs during embryogenesis is diverse. Members of the *HOX* gene cluster are required for maintenance and self-renewal of hematopoietic progenitors or HSCs [143, 150]. Expression of the *HOX* gene clusters is controlled by upstream regulators, such as the MLL [142], members of the CDX TFs (CDX1, CDX2, and CDX4) [97] or the retinoic acid signaling pathway [151]. Dysregulation of the *HOX* TFs was associated with different hematopoietic malignancies [148], reflecting the crucial role and complexity of regulation of the *HOX* gene cluster. *Hox* knockout studies validated the crucial role of their function during hematopoietic ontogeny and HSC maintenance. Especially *HoxA9* knockouts demonstrated severely impaired HSC self-renewal and proliferation [152] and significantly decreased the reconstitution capacities of fetal liver HSCs in mice [153]. It was hypothesized that the medial *HOXA* genes have a key role during hematopoietic differentiation and that the lack of *HOXA* expression might be a significant barrier that prevents the in vitro generation of human PSC-derived bona fide HSCs [151]. Several gain-of-function studies validated the crucial role of *HOX* genes. Ectopic expression of *HOXA9* alone was insufficient to confer self-renewal or long-term repopulation potential to human ESC-derived HPCs [154]. A different approach identified crucial TF combinations to overcome erythro-myeloid restriction and confer enhanced, HSC-like properties to human PSC-derived hematopoietic cells [155]. An extensive in vitro screen identified the TF combination HOXA9, ERG and RORA to be sufficient to respecify the myeloid restricted, PSC-derived CD34^+^/CD38^−^ HPCs to a proliferative, self-renewing stage with an enhanced erythroid and lymphoid lineage potential. The addition of *SOX4* and *MYB* overexpression enabled short-term myelo-erythroid engraftment. Although ectopic expression of these TFs enhanced the stem cell properties of the formerly restricted HPCs, long-term engraftment and multi-lineage reconstitution were not achieved. Interestingly, it was hypothesized that the definitive hematopoietic program and HSC generation in vitro might be actively repressed through epigenetic silencing [156]. A screening experiment for DNA- and histone-modifying factors that repress the definitive hematopoietic program and multipotency identified EZH1 as a crucial repressor. EZH1 is a component of the Polycomb repressive complex 2 and mediates target-site-specific epigenetic silencing through histone methylation. Strikingly, EZH1 was found to directly bind promoters of HSC-associated genes, such as *HLF*, *HOPX*, *MEIS1*, *PRDM16*, *LMO2*, *ETS1*, *HES1*, *RUNX1* and *HOX* clusters. *EZH1* knockdown increased gene expression of arterial- and HSC-associated genes such as *NOTCH*, *HES1*, *HEY1*, *SOX17*, *RUNX1T1* and *FOXC2*, and elicited robust T and B cell potential of the previously described [155] differentiation protocol [156]. In mice, Ezh1 deficiency or haploinsufficiency increased the HSC frequencies compared to wild type animals, and stimulated the precocious generation of bona fide HSCs during in vivo ontogenesis, presumably through enhanced accessibility of key HSC TF-binding sites [156]. A combined approach that applied directed differentiation and TF-mediated specification was shown to confer HSC-like functionality to human PSC-derived HE [116]. A library of 26 fetal liver HSC-enriched TFs was used to screen for a factor combination to confer HSC functionality to a PSC-derived HE population. The CD34^+^/KDR^+^/CD43^−^/CD235a^−^ endothelium was transduced with this library and 24 h later intrafemorally injected into sublethally irradiated mice. Multi-lineage engraftment of myeloid, erythroid and lymphoid lineages was observed 12 weeks post-transplantation. Enrichment of the seven TFs *HOXA5*, *HOXA9*, *HOXA10*, *ERG*, *LCOR*, *RUNX1* and *SPI1* was consistently detected, indicating that these factors enabled self-renewal, engraftment and multi-lineage reconstitution potential [116]. Engraftment of secondary recipient mice validated the self-renewal capacity that was conferred by the 7 TFs. However, compared to cord blood CD34^+^-transplanted mice, the robustness of the multi-lineage engraftment (9/76 mice) was lower and also the full recapitulation of the reconstituted lineages was biased [116]. This approach suggested that the generation of PSC-derived bona fide HSC is becoming more feasible. However, TF-based strategies for the in vitro generation of PSC-derived, bona fide HSCs for clinical use remains a high priority that has yet to be realized.

## Conclusion and perspectives

In summary, recent work clearly illustrated remarkable progress in the conversion of (i) PSCs and somatic cell types into HE as an important intermediate towards the development of HSCs. As the generation of fully engraftment-competent HSCs with multi-lineage developmental capacity in the sense of definitive hematopoiesis is cumbersome and a goal that remains to be achieved, we can further learn from the natural development of HE, subsequent HSCs and their neighboring niche components to identify crucial extrinsic and intrinsic regulating factors. Here, insights in single cell transcriptomics, including scRNAseq, will continue to identify critical developmental steps and cell types and will shed further light on the underlying transcriptional network, including instructive TFs and their expression levels. Moreover, spatial transcriptomics may further unravel the role of neighboring cells, including the role of crucial components of the microenvironment, necessary for conferring HSC identity, functionality, maintenance and expansion.

Correct dosing and timing of expression of transcription factors and extrinsic niche factors will be important to mimic and recapitulate the complex developmental process in vitro, for which state-of-the-art vector systems for regulated, timed and dosed expression will be needed. Here, especially transient vector expression systems will be interesting to explore the possibilities to mimic the waves and levels of hematopoietic factor expression. In addition, transient expression patterns will be desirable to avoid permanent expression of potentially oncogenic TFs and growth factors, and thus reduce (pre)malignant transformation of hematopoietic progenitors. For example, controlled delivery of the necessary TFs at the optimal time window during differentiation could be a further improvement of direct conversion protocols and forward programming strategies.

Although enforced overexpression of TFs has been used to increase hematopoietic potential and functionality of in vitro-derived hematopoietic cells, clinical translation of TF-based approaches remains to be achieved due to insufficient functionality and quantity of the cell product. The knowledge gained from TF-based strategies is helping to elucidate the key regulatory pathways whose modulation is necessary for directed differentiation towards HSCs. Future strategies will exploit this information to generate bona fide HSCs without the potential dangers of transformation due to TF overexpression.

Looking into the future, while we are getting closer to being able to generate high-quality transplantable hematopoietic cells, it will be necessary to establish the framework for GLP-/GMP (good laboratory practice/good manufacturing practice)-compliant production, including the generation of standard operating procedures (SOPs) and the inclusion of fully traceable and animal-free reagents in a GLP-compatible lab environment, to create a perspective for upscaling as needed in future clinical trials. For example, GMP-compliant cell modification and TF delivery strategies will have to be developed.

Moreover, thinking in the context of next-generation hematopoietic cell transplants, the horizon of combined gene and cell therapeutics should be considered. The use of precision medicine approaches, e.g., clinically used viral vectors and next-generation genome editing tools, will allow the tailored repair of genetic defects of autologous transplants as well as the generation of allogeneic “off-the-shelf” transplants, which may be transplantable to a broad spectrum of patients and diseases, especially in cases in which no suitable HSC donor is available. In addition to HSCs, also PSC-derived T, NK and NKT cells are interesting tools for tailored immunotherapeutics. HLA borders represent an important bottleneck to allogeneic cell replacement strategies. Here, the use of biobanking of iPSC for frequently used HLA subtypes or other “off-the-shelf” implementation strategies could be helpful.

Taken together, the increasing insights in PSC-derived hematopoiesis as well as the HE may allow the tailored generation of hematopoietic cells for disease modeling, cell therapy and potentially even next-generation transplants.
